# Procalcitonin and C-Reactive Protein/Procalcitonin Ratio as Markers of Infection in Patients With Solid Tumors

**DOI:** 10.3389/fmed.2021.627967

**Published:** 2021-03-12

**Authors:** Matteo Vassallo, Celine Michelangeli, Roxane Fabre, Sabrina Manni, Pierre L. Genillier, Nicolas Weiss, Elea Blanchouin, Laurence Saudes, Regis Kaphan, Annick Puchois, Christian Pradier, Nathalie Montagne

**Affiliations:** ^1^Department of Internal Medicine/Infectious Diseases, Cannes General Hospital, Cannes, France; ^2^Department of Public Health, L'Archet Hospital, University of Nice, Nice, France; ^3^Department of Medical Informatics, Cannes General Hospital, Cannes, France; ^4^Multipurpose Laboratory, Bacteriology and Virology Unit, Cannes General Hospital, Cannes, France

**Keywords:** procalcitonin, C-reactive protein/procalcitonin ratio, fever, infection, solid tumor

## Abstract

**Objectives:** The roles of procalcitonin (PCT) and C-reactive protein (CRP) in febrile cancer patients is currently unclear. Our aim was to assess these in febrile patients with solid tumors and to identify cut-off values for ruling out infection.

**Methods:** We retrospectively evaluated patients with solid tumors admitted to hospital due to fever. They were divided into those with Fever with microbiologically documented infection (FMDI), Fever with clinically documented infection (FCDI) and Tumor-related fever (TRF). PCT and CRP levels were compared. Receiver-operating curves were plotted to define the best cut-off values for discriminating between infection-related and cancer-related fever.

**Results:** Between January 2015 to November 2018, 131 patients were recorded (mean age 68 years, 67% male, 86% with metastasis). Patients with FMDI or FCDI had significantly higher baseline levels of PCT and lower CRP/PCT than those with TRF. A PCT cut-off value of 0.52 ng/mL for discriminating between infection and cancer-associated fever yielded 75% sensitivity, 55% specificity, 77% positive predictive value (PPV), and 52% negative predictive value (NPV). A CRP/PCT ratio with a cut-off value of 95 showed 56% sensitivity, 70% specificity, 79% NPV, and 44% PPV.

**Discussion:** PCT is a sensitive marker of sepsis or localized infection in patients with solid tumors, but its specificity is poor. The CRP/PCT ratio improves specificity, thus providing a reliable means of ruling out infection for values above 95.

## Introduction

Treating patients with cancer often requires managing systemic and localized infection, which is associated with significant morbidity and mortality (1). However, fever in oncologic patients is a non-specific clinical marker, which may be related to genuine infection, but also to other clinical conditions, i.e., paraneoplastic syndrome or chemotherapy. Cancer patients admitted to hospital for fever thus require rapid and appropriate decisions for survival. Laboratory tests can be useful for clinical decisions in the initial assessment. Among these, abnormal leucocyte count and C-reactive protein (CRP) are highly non-specific and generally do not distinguish between an infectious and a non-infectious etiology. Among the most promising and specific biomarkers of infection, procalcitonin (PCT) has been reported as a reliable indicator of bacterial, fungal and protozoal infections (2–4). It is usually rapidly produced in liver, lung, kidney and other tissues from the onset of infection. In healthy subjects its values are low (<0.1 ng/ml) and the cut-off value of 0.5 ng/ml is generally used for ruling out bloodstream infections (5). However, as cancer cells may increase pro-inflammatory cytokine expression, the specificity of PCT in cancer patients is generally poorer than for other patients. Indeed, the clinical interpretation of PCT in these patients has been evaluated in several studies and results are contradictory (6–14), requiring further investigation of the role of current biomarkers to discriminate between the infectious and non-infectious origin of fever in cancer patients. The aim of this study was to retrospectively study the reliability of PCT and CRP in cancer patients admitted for fever and to identify the appropriate cut-off values for ruling out infection. As PCT values may differ significantly between solid tumors and hematological malignancies, usually increasing the most among the latter, we decided to restrict our analysis to patients with solid tumors.

## Methods

### Participants

Using the database of Cannes General Hospital, we extracted data from patients admitted to the Department of Internal Medicine for fever and active solid tumors from January 2015 to November 2019, with available PCT measurements at symptoms onset. Data were collected from electronic medical records and included: age, gender, underlying cancer and stage thereof, comorbid conditions, vital signs, imaging data, microbiological data and laboratory markers. Among laboratory markers, leucocyte count, CRP, PCT and CRP/PCT upon admission were collected.

### Diagnostic Criteria

Each file was reviewed in order to confirm the final diagnosis and the etiology of fever. In particular, microbiological, radiological and clinical data were reviewed and physicians who had been directly involved in the care of patients took part in this analysis.

According to the final diagnosis, patients were divided into the following groups:

- Fever with microbiologically documented infection (FMDI), when a causative pathogen was isolated from microbiological cultures. Bacteremia was considered when one positive culture was obtained, with the exception of coagulase-negative Staphylococci, which required at least two positive blood cultures to confirm bacteremia.
- Fever with clinically documented infection (FCDI), when clinical and/or radiological signs were in favor of infection, but microbiological cultures were negative.
- Tumor-related fever (TRF), if patients had no microbiological, radiological or clinical evidence of infection and the final diagnosis was fever of non-infectious origin.

### Statistical Analysis

Patients with FMDI and those with FCDI were compared with those with TRF. First, we described demographic, clinical, radiological and microbiological data and laboratory markers in both groups using univariate logistic regression. In case of factors with univariate *p*-values < 0.2, multivariate models were then fitted.

In case of significant differences in PCT and CRP/PCT markers between groups, sensitivity, specificity, positive predictive value (PPV) and negative predictive values (NPV) were measured and receiver-operating-characteristic (ROC) curves were plotted to identify the best cut-off values.

As care of patients with bloodstream infection is particularly critical and the need for better markers is crucial, we then stratified patients according to those with positive and negative blood cultures.

## Results

### Population Characteristics

Among 5,883 patients admitted to the Department of Internal Medicine from January 2015 to November 2018, 131 were febrile and had active solid tumors, with PCT measurements available upon admission (mean age 68 years, 67% male, 86% with metastatic cancer, 66% receiving chemotherapy, 36% with gastro-intestinal cancer, 21% with genitourinary neoplasia, see Table 1). Only nine out of 131 patients received antibiotics for a short period just before admission.

**Table 1:** Patient characteristics.

	***N* (%) or mean [SD]**
Number of patients	131
Male gender	88 (67.2%)
Age (years)	67.9 [12.4]
**Underlying cancer**	
Colorectal	25 (19.0%)
Other gastrointestinal cancer	23 (17.6%)
Genitourinary	28 (21.4%)
Lungs	16 (12.2%)
Gynecological	17 (13.0%)
Oral cancer	11 (9%)
Other	11 (8.4%)
**Cancer stage**	
Metastatic	112 (86.2%)
Locally advanced cancer	18 (13.8%)
Undergoing chemotherapy	86 (66.0%)
Severe neutropenia (<500/microliter)	2 (1.5%)

### Clinical Groups and Laboratory Markers

According to the final diagnosis, 66 patients had microbiologically documented infection (51%), 21 clinically documented infection (16%) and 44 tumor-related fever (33%). Among the 66 patients with FMDI, 47 had bloodstream infection (72%), while six had upper urinary tract infection and five gastro-intestinal infection.

Among the 21 patients with FCDI, the majority had respiratory tract infection (10 patients, 48%).

Only two out of 131 subjects had severe neutropenia upon admission, defined as a white cell count below 500 cells per microliter.

Patients with microbiologically or clinically documented infection had significantly higher baseline levels of PCT and lower levels of CRP/PCT than subjects with TRF (see Table 2).

**Table 2:** Comparison between patients with infection-related and tumor-related fever.

	**FMDI or FCDI**	**TRF**	
	***N* (%) or** ** mean [SD]**	***N* (%) or** ** mean [SD]**	***p*-value**
Number of patients	87	44	
**Clinical diagnosis**			
FMDI	66 (76%)	–	–
FCDI	21 (24%)	–	–
TRF	–	44 (100%)	–
Male gender	61 (70%)	27 (61%)	0.314
Age (years)	69.2 [12.3]	65.4 [12.5]	0.103
Metastatic cancer	69 (80%)	43 (98%)	**0.006**
White cell count below 500/mm^3^	2 (2%)	0 (0%)	0.550
Antibiotic therapy prior to admission	8 (9%)	1 (2%)	0.163
Baseline C-reactive protein value (mg/L)	164.9 [111.5]	164.0 [115.3]	0.981
Baseline procalcitonin value (ng/mL)	11.3 [23.4]	1.7 [3.3]	**0.001**
Baseline CRP/PCT ratio	280.4 [641.2]	630.9 [1,001.2]	**0.001**

*FMDI, Fever with microbiologically documented infection; FCDI, Fever with clinically documented infection; TRF, Tumor-related fever*.

*Values in bold mean for statistically significant values*.

**Figure 1:**
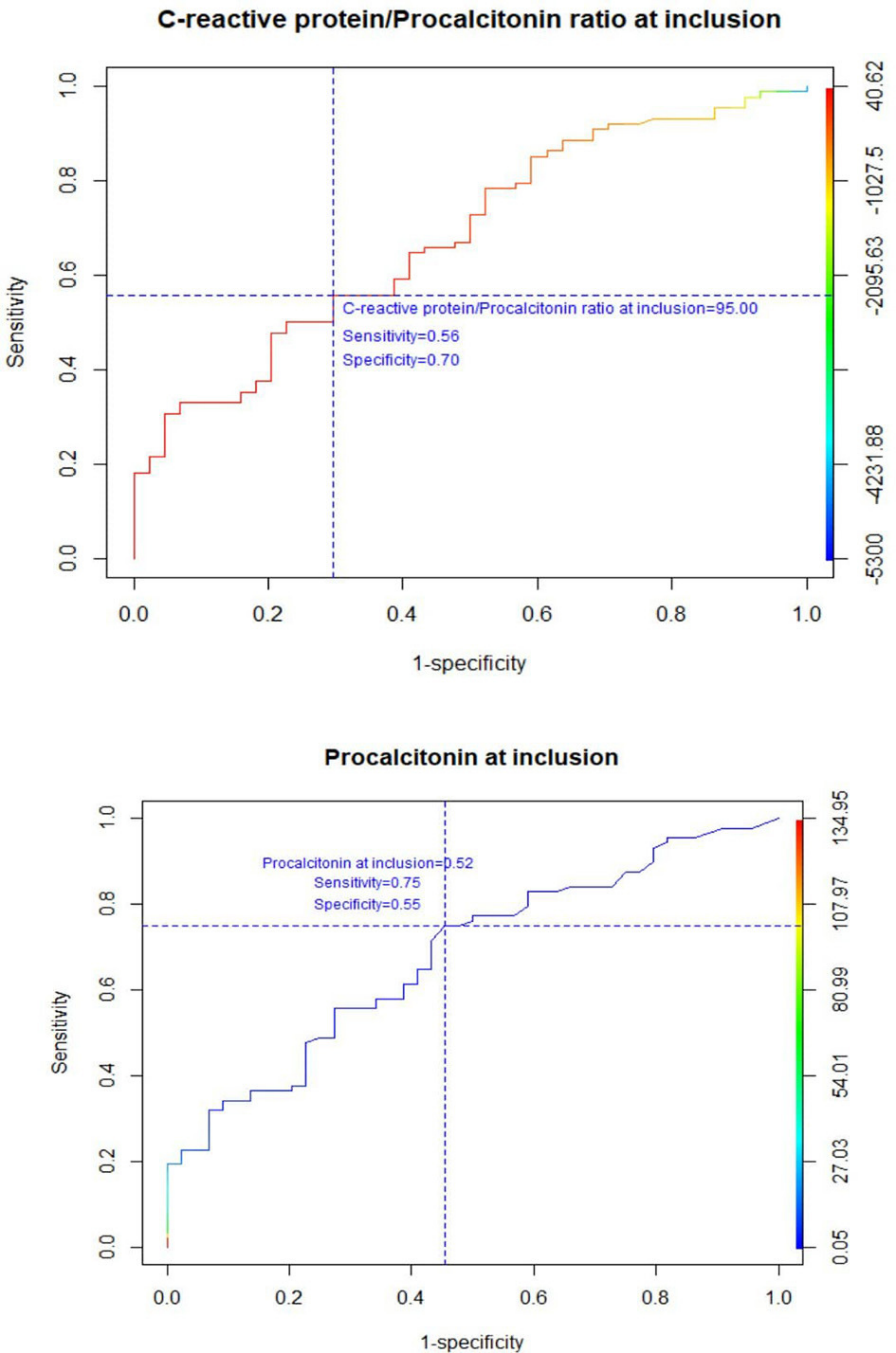
Receiver operating characteristics curve for CRP/PCT ratio and PCT at inclusion.

The ROC analysis demonstrated that the PCT cut-off value of 0.52 ng/mL was associated with a sensitivity of 75%, a specificity of 55%, a PPV of 77%, and a NPV of 52% for discriminating between infection-related and cancer-related fever (Figure 1). Further, a ROC curve for a cut-off value of the CRP/PCT ratio was plotted to improve the specificity, showing that a value of 95 was associated with high specificity (70%) and negative predictive value (79%), but poor sensitivity (56%), and PPV (44%, Figure 1).

Neither neuroendocrine cancer nor metastatic lesions, which are potential factors for increasing PCT concentrations regardless of infections (1), were associated with significantly different PCT values (see Supplementary Materials).

Moreover, as immune therapy could represent another potential interfering factor for the interpretation of PCT, we compared subjects who were receiving immune therapy with the others, and we found no difference in terms of PCT values at baseline (see Supplementary Materials).

### PCT and CRP/PCT Levels in Bloodstream Infections

Comparison between patients with bloodstream infection and those with negative blood cultures showed that baseline PCT and CRP/PCT levels were also significantly different (Table 3).

**Table 3:** Comparisons between patients with and without positive blood culture.

	**Positive blood cultures**		
	**No-*n* = 80**	**Yes-*n* = 47**	
	**Mean [SD]**	**Mean [SD]**	***p*-value**
Age	67.3 [12.1]	69.2 [13.1]	0.304
Leucocyte cell count (cc/mm^3^)	11,473.6 [6,064.8]	11,084.3 [9,599.1]	0.179
CRP at the admission	159.0 [100.3]	183.5 [129.7]	0.472
PCT at the admission (ng/ml)	4.8 [13.8]	14.3 [26.4]	**0.023**
CRP/PCT	455.3 [810.8]	306.7 [796.3]	**0.029**
	***n*** **(%)**	***n*** **(%)**	***p*****-value**
**Gender**			0.212
Female	29 (36.3)	12 (25.5)	
Male	51 (63.8)	35 (74.5)	
**Antibiotic treatment at home**			1.000
No	73 (93.6)	43 (93.5)	
Yes	5 (6.4)	3 (6.5)	
**Metastatic cancer**			**0.024**
No	7 (8.9)	11 (23.4)	
Yes	72 (91.1)	36 (76.6)	
**Severe neutropenia**			0.530
No	78 (97.5)	47 (100.0)	
Yes	2 (2.5)	0 (0.0)	

*CRP, C-reactive protein; PCT, Procalcitonin*.

*Values in bold mean for statistically significant values*.

No difference in either PCT or CRP/PCT levels was found between gram- positive and gram-negative sepsis (data not shown).

## Discussion

In patients with solid tumors admitted to hospital for pyrexia, we showed that the combination of baseline PCT and CRP/PCT ratio measurements display good performance for discriminating between infection-related and cancer-related fever. This could be particularly helpful for guiding clinicians' appropriate decisions, even in patients with advanced cancer.

Our study confirms that PCT is a sensitive marker of sepsis or localized infection in this patient population, but its specificity is poor (15). Indeed, in cancer patients, values of PCT might be elevated regardless of infection, e.g., as a consequence of metastasis, neuroendocrine function of malignant cells (1) or production of pro-inflammatory cytokines (16). Our work also confirms that a CRP measurement alone is less useful than PCT, due to its nonspecific character (17).

Adding the measurement of the CRP/PCT ratio significantly improved specificity, thus showing good performance in ruling out infection as the cause of fever for values above 95. If confirmed by larger studies with a prospective design, the key message could be that the wider the difference between CRP and PCT values, the higher the chances for a tumor-related origin of fever. Both CRP and CRP/PCT could therefore be helpful in guiding decisions in a two-stage decision-making process: a PCT value above 0.5 points to potential infection. However, a CRP/PCT value above 95 is in favor of cancer-related fever.

As expected, PCT and CRP/PCT values were significantly different in patients with bacteremia compared to those with negative blood cultures. Indeed, patients with bloodstream infection generally have higher PCT serum concentrations (18).

As the lipopolysaccharide is the major component of gram-negative bacteria and is responsible of TNF-alpha guided PCT production, PCT is generally viewed as a useful marker for identifying gram-negative sepsis. In contrast, in our study there was no difference between gram-positive and gram-negative sepsis in terms of baseline PCT values. This result needs to be investigated through larger prospective studies, but it could be due to the lipoteichoic acid molecules on the gram-positive cell wall triggering the inflammatory response (1).

The major limitation of this study, together with the relative small number of patients included, rests on its retrospective character. Only prospective studies including PCT and CRP/PCT values at inclusion could determine the role of these markers in guiding clinical decisions. Considering fever in patients as due to infection when lacking microbiological confirmation could be another limitation. However, files were all reviewed in order to confirm the infectious etiology. Moreover, half of them had clear radiological signs of pneumonia. Besides, only two patients had severe neutropenia, which is known to be a heterogeneous clinical entity (19). Finally, further prospective studies, including also other types of tumors are necessary for better assessing the role of PCT and CRP in clinical decisions.

In conclusion, PCT and CRP/PCT values upon admission could assist in diagnosing infection in patients with solid tumors and should be considered in the decision-making process regarding their clinical management. Further studies on the role of PCT in patients with hematological malignancies would also be useful.

## Data Availability Statement

The original contributions presented in the study are included in the article/Supplementary Material, further inquiries can be directed to the corresponding author/s.

## Ethics Statement

The studies involving human participants were reviewed and approved by French National Institute for Health Data (Institut National de Données de Santé). The patients/participants provided their written informed consent to participate in this study.

## Author Contributions

MV, PG, CM, SM, and NW: conceived, designed the study and collected data. MV: analyzed the data and wrote the manuscript. EB, LS, RK, and NM: edited the manuscript. All authors contributed to the article and approved the submitted version.

## Conflict of Interest

The authors declare that the research was conducted in the absence of any commercial or financial relationships that could be construed as a potential conflict of interest.
