# Supplementary material for: Procalcitonin and C-Reactive Protein/Procalcitonin Ratio as Markers of Infection in Patients With Solid Tumors
Source: Front Med (Lausanne). 2021 Mar 12;8:627967. doi: 10.3389/fmed.2021.627967 (PMC7994271; doi:10.3389/fmed.2021.627967)
Supplement: Supplementary file 1 [file Table_1.DOCX]

Supplementary materials:

Procalcitonin and C-reactive protein values according to other potential interfering factors

|  | Metastatic lesions | | | |  |
| --- | --- | --- | --- | --- | --- |
|  | No – n=18 | | Yes – n=113 | |  |
|  | Mean | [SD] | Mean | [SD] | p-value |
| PCT | 9.0 | [16.7] | 7.7 | [19.9] | 0.306 |
| CRP/PCT | 123.2 | [64.3] | 161.1 | [112.4] | 0.260 |
|  | Current immune therapy | | | |  |
|  | No – n=113 | | Yes – n=10 | |  |
|  | Mean | [SD] | Mean | [SD] | p-value |
| PCT | 7.6 | [19.9] | 10.8 | [17.7] | 0.101 |
| CRP/PCT | 156.3 | [106.5] | 148.8 | [84.7] | 0.959 |
|  | Neuroendocrine cancer | | | |  |
|  | No – n=126 | | Yes – n=5 | |  |
|  | Mean | [SD] | Mean | [SD] | p-value |
| PCT | 8.0 | [19.7] | 10.8 | [20.9] | 0.601 |
| CRP/PCT | 152.9 | [103.8] | 247.0 | [158.2] | 0.117 |

CRP : C-reactive protein

PCT : Procalcitonin
